# Derivation of Patient Specific Pluripotent Stem Cells Using Clinically Discarded Cumulus Cells

**DOI:** 10.1371/journal.pone.0165715

**Published:** 2016-11-01

**Authors:** Wei-Fang Chang, Yuh-Ming Hwu, Jie Xu, Chen-Ju Lin, Sheng-Wen Wang, An-Sheng Cheng, Jean Lu, Chung-Hao Lu, Li-Ying Sung

**Affiliations:** 1 Institute of Biotechnology, National Taiwan University, Taipei, Taiwan; 2 Department of Obstetrics and Gynecology, Mackay Memorial Hospital, Taipei, Taiwan; 3 Department of Obstetrics and Gynecology, Mackay Medical College, New Taipei City, Taiwan; 4 Department of Obstetrics and Gynecology, Mackay Junior College of Medicine, Nursing, and Management, Taipei, Taiwan; 5 Center for Advanced Models for Translational Sciences and Therapeutics, University of Michigan Medical Center, Ann Arbor, Michigan, United States of America; 6 Yupintang Traditional Chinese Medicine Foundation, Kaohsiung, Taiwan; 7 Genomics Research Center, Academia Sinica, Taipei, Taiwan; 8 Center for Biotechnology, National Taiwan University, Taipei, Taiwan; 9 Animal Resource Center, National Taiwan University, Taipei, Taiwan; 10 Agricultural Biotechnology Research Center, Academia Sinica, Taipei, Taiwan; Harvard Medical School, UNITED STATES

## Abstract

Induced pluripotent stem cells (iPSCs) are powerful tools for basic and translational research, as well as regenerative medicine. In routine human *in vitro* fertilization (IVF) practices, cumulus cells (CCs) are discarded, representing a potential source of biological materials for regenerative medicine. In this study, we derived patient-specific iPSCs using CCs from human infertility clinics for the first time. The human cumulus cell derived iPSCs (hc-iPSCs) were characterized for growth, karyotype, expression of pluripotency genes, and were subjected to embryoid bodies (EBs) and teratoma assays to evaluate their differentiation capacity. Hc-iPSCs display typical iPSC characteristics, and are capable of differentiating into all germ layers *in vitro* and *in vivo*. We further show that putative primordial germ cell like cells (PGCLCs) can be derived using hc-iPSCs. Our data demonstrate the feasibility of deriving patient-specific pluripotent stem cells using CCs.

## Introduction

The induced pluripotent stem cell (iPSC) technology is one of the most important breakthroughs in regenerative medicine in the past decade [1–3]. Using this technology, researchers can directly convert somatic cells to pluripotent states, bypassing the need for human embryos and therefore avoiding related ethical, religious and legal concerns that are associated with human embryonic stem cells (ESCs) [4–6]. Further, iPSCs can be patient-specific, therefore have the potential to solve the immunological compatibility issues. With the technology advancement, iPSCs can now be derived using versatile methods including both integrating and non-integrating means, and from different donor cells such as fibroblast cells, epithelia cells, hepatocytes, and circulating blood cells [7–13].

Cumulus cells (CCs) surround the oocyte both in the ovarian follicle and after ovulation. CCs originate from granulosa cells (GCs) which differentiate into mural GCs and CCs during follicular antrum formation [14]. CCs are discarded in routine human intracytoplasmic sperm injection (ICSI) practices. Historically, CCs were among the first cell types used for animal cloning and have been widely used for nuclear transfer (NT) based reprogramming studies [15,16]. Surprisingly however, there is little work on the derivation of human iPSCs using CCs. In one study, pooled GCs, but not CCs, collected from follicle fluid, were used to establish human iPSC lines successfully [17]. In another study, the only one to date to our knowledge, human iPSCs were established using CCs [18]; however these pooled cells are not patient specific, and it is not known whether these cells are capable of differentiating to germ cells.

The aim of this study is to prove that clinically discarded CCs can be utilized to derive patient-specific iPSCs (hc-iPSCs), and once established, to evaluate their differentiation capacity *in vitro* and *in vivo*. We are also interested in whether hc-iPSCs can be used for gamete derivation, considering the close physical and physiological relationship between the CCs and the oocyte. Our work has implications in human reproduction and regenerative medicine.

## Materials and Methods

All reagents were purchased from Thermo Fisher Scientific (Waltham, MA, USA) unless otherwise indicated.

### Clinically Discarded CCs

The work is approved by Institutional Review Boards (IRB) at Mackay Memorial Hospital (MMH), IRB approval number: 14MMHIS261, and the Institutional Animal Care and Use Committee (IACUC) of National Taiwan University (NTU), IACUC approval number: NTU-103-EL-92. All the participants in this study understand the research and the written consent forms have been obtained from the patients. Patients were subjected to the controlled ovarian hyperstimulation (COH) treatments at MMH using the GnRH antagonist protocol as described [19]. Clinically discarded CCs were collected with patients’ consent. Basic information of the patients is shown in Table 1. Freshly collected CCs from the same patient were pooled and immediately plated on matrigel (354234, Corning, NY, USA) coated 96-well dish in culture medium containing 2% fetal bovine serum (FBS, 16000–044), 0.05 mM ascorbic acid (A4403, Sigma-Aldrich, St. Louis, MO, USA), 0.05 μM dexamethasone (D4902, Sigma), 20 ng/mL epidermal growth factor (EGF, E9644, Sigma), basic fibroblast growth factor (bFGF, 13256–029, 50 ng/mL) and 1U/mL follicle stimulating hormone (FSH, F2293, Sigma). Medium was changed every 2–3 days after the cells attached on the dish. Twenty days post seeding, confluent cells were trypsinized by 0.05% Trypsin-EDTA. The cultured CCs at Passage 2 were subjected for hc-iPSC derivation.

**Table 1:** Summary of patient information.

Patient	Age	Reason for treatment	AMH* (ng/mL)	Number of eggs retrieved	Number of matured MII eggs	Number of eggs fertilized (ICSI)	Pregnancy outcome	Number of iPSC line established
Patient#1	36	Ovulation dysfunction	1.06	3	3	3	No	4
Patient#2	34	Ovulation dysfunction	1.4	8	5	2	Yes	0
Patient#3	38	Male factor	0.84	3	2	1	No	0
Patient#4	44	Ovulation dysfunction	6.13	20	19	19	All embryos were frozen	0
Patient#5	36	Male factor	1.15	4	3	3	No	4
Patient#6	34	Uterus factor	2.47	11	8	6	No	0
Patient#7	38	Male factor	0.84	17	14	13	No	0

*AMH: Anti-Müllerian Hormone.

### Derivation and Maintenance of hc-iPSCs

The lentiviral cDNA plasmids of human *OCT4*, *SOX2*, *NANOG* and *LIN28* (OSNL) were obtained from Addgene (ID: 16580, 16578, 16577, 16579) [20], and the transgene-expressing lentivirus was produced in 293T cells [20,21]. CCs (2x10^4^ per 24-well) were seeded one day before transduction. A multiplicity of infection (MOI) of 5 was used for transduction. Cumulus cell culture medium was used during seeding and transduction, and for 15 days following transduction, after which conventional human ESC culture medium (hESM) containing 20% Knockout Serum Replacement (KSR, 10828–028), 2 mM Glutamax (35050–061), 0.1mM 2-mercaptoethanol (ES-007-E, Millipore, Billerica, MA, USA), 0.1 mM nonessential amino acids (NEAA, 11140–050), and 4 ng/mL bFGF in DMEM/F12 medium (11330–032) was used in the same well. Putative iPSC colonies were manually picked up with a fire-polished glass Pasteur pipette and plated on mitomycin C (2μg/mL, M4287, Sigma) treated E13.5 mouse embryonic fibroblasts (MEF) around 25 days post transduction. After one week, colonies grew to about 1.5 mm in diameter and were then treated with dispase (1 mg/mL, 04942086001, Roche Applied Science, Penzberg, Upper Bavaria, Germany) for passaging. The human female embryonic stem cell H9 (WiCell Research Institute, Inc., Madison, WI, USA), female (iPSC0102) and male (iPSC0207) iPSC lines (Human Disease iPSC Service Consortium, Taipei, Taiwan) derived from peripheral blood mononuclear cells (PBMCs), hereafter referred to as hb-iPSC lines, using CytoTuneTM-iPS 2.0 Sendai reprogramming Kit (A16517), were used as control in this study, also maintained in hESM with MEF feeders and passaged following the same procedure.

### Growth Curve

2x10^5^ cells were plated in each well of a 6-well dish, and cells were collected and counted on days 2, 4 and 6. Population doubling time was analyzed using an online tool (http://www.doubling-time.com/compute.php).

### Immunofluorescent Staining

The putative hc-iPSCs were fixed with 4% paraformaldehyde (PFA) in DPBS for 20 minutes for immunofluorescent staining, following our routine protocol [22]. Primary antibodies against OCT4 (1:150, MAB4401, Millipore), SOX2 (1:150, GTX101507, Genetex, Hsinchu, Taiwan), SSEA4 (1:150, MAB4304, Millipore), TRA1-60 (1:200, MAB4360, Millipore) and TRA-1-81 (1:200, MAB4381, Millipore) were used for detecting pluripotency, and those against SOX17, BRACHYURY (1:50. AF1924 and AF2085, R&D Systems Inc., Minneapolis, MN, USA) and β-III-TUBULIN (or TUJ1, 1:200, MAB1637, Millipore) were used for detecting germ layers differentiation. Secondary antibodies include Alexa Fluor goat anti mouse 488 (A11029), goat anti rabbit 488 (A11008), and donkey anti mouse IgM Cy3 (715-165-140, Jackson ImmunoResearch Inc., West Grove, PA, USA). Fixed samples were also subjected to alkaline phosphatase (AP) detection by VECTOR Blue Alkaline Phosphatase Substrate Kit (SK-5300, Vector Laboratories, Burlingame, CA, USA). Teratoma tissues were dissected and immersed in 4% PFA overnight at 4°C and then embedded into wax. Sections were dewaxed, rehydrated and stained with hematoxylin and Eosin (H&E).

### Flow Cytometry

The hc-iPSCs were dissociated by 0.05% Trypsin-EDTA and washed with D-PBS supplemented with 2% FBS (FACS solution). For antibody dilution and washing we used FACS solution in further steps. Dissociated cells were incubated with primary antibody, SSEA4 and TRA-1-60 (1: 200), for 30 minute on ice. After washing, cells were incubated with secondary antibodies with donkey anti mouse IgM Cy3 and Alexa Fluor goat anti mouse 488 (1: 200) for another 30 minutes. Finally, cells were washed and analyzed on a flow cytometer Cytomic FC 500 (Beckman Coulter, Inc., Brea, CA, USA).

### Karyotyping

Karyotyping was carried out in Department of Obstetrics and Gynecology of Mackay Memorial Hospital. 15–30 cell clumps of hc-iPSCs were manually picked-up to matrigel-coated coverslips from growing colonies and maintained in hESM for 1 day. Cells were then treated with Colcemid (0.1 mg/mL, 15210040) for 3 hours and DNA was stained with Wright stain for 3 minutes. Thirty chromosome spreads from each iPSC line was analyzed by Applied Spectral Imaging BandView (Applied Spectral Imaging, Inc., Carlsbad, CA, USA).

### *In Vitro* Differentiation

EBs were obtained from hc-iPSCs by routine passage and transferred to 60 mm petri-dish for suspension culture in hESM without bFGF for 10 days. EBs were then plated on matrigel-coated dish in KO-DMEM medium containing 20% FBS to allow spontaneous mesoderm and endoderm differentiation for 5 to 7 days. For neuronal (representing ectoderm) differentiation, EBs from hc-iPSCs were switched to N2 medium containing DMEM/F12, 0.1mM NEAA, 2 mM Glutamax, 1X N2 supplement (17502–048), 1 mM sodium pyruvate (11360–070), and bFGF (20 ng/mL) for 3 days, then attached to matrigel for neurite growth in N2 medium for 18 days. Attached EB samples were fixed for immunofluorescent staining and 10-days-EBs were collected for quantitative reverse transcription-polymerase chain reaction (qRT-PCR) to confirm germlayer markers.

### Teratoma Assay

Hc-iPSCs were cultured in hESM supplemented with 10 μM ROCK inhibitor Y27632 (04–0012, Stemgent, Inc., Lexington, MA, USA) one day before they were transplanted. On Day 0 (i.e. transplantation day), cells were trypsinized and filtered with cell strainer (352235, Corning), after which 1x10^6^ cells were injected intramuscularly into a 6–8-week-old female NOD-SCID mouse (BioLASCO, Taipei, Taiwan). Terotomas were collected 6 weeks post transplantation.

### Induction of PGCLCs from hc-iPSCs

We followed a published protocol [23] to test the feasibility of differentiating hc-iPSCs to primordial germ cell-like cells (PGCLCs) *in vitro*. Prior to PGCLC induction, hc-iPSCs, hb-iPSCs (iPSC0102, iPSC0207) and hESC H9 were cultured in 4i hESM medium for 3 passages. The 4i hESM is based on the hESM medium, with the following additions: 3 μM CHIR99021 (Axon 1368. Axon Medchem BV, Groningen, Netherlands), 1 μM PD0325901 (Axon 1408, Axon), 5 μM SB203580 (1202, Tocris Bioscience, QL, United Kingdom), and 5 μM SP600125 (1496, Tocris), TGF-β1 (1 ng/ml, 100–21, Peprotech, NJ, USA), human LIF (20 ng/ml, 300–05, Peprotech), and a higher concentration of bFGF (8 ng/mL).

Upon induction, cells were treated with TrypLE express (12605–036), then plated to ultra-low attachment U-bottom 96-well plate in primordial germ cell (PGC) induction medium, which contained 15% KSR, 0.1 mM NEAA, 0.1 mM 2-mercaptoethanol, 1X Penicillin and Streptomycin (15070–063), 2 mM Glutamax, 1 mM Sodium pyruvate, and the following cytokines: 250 ng/ml BMP4 (314-BP-050, R&D), 250 ng/ml BMP2 (355-BM-050, R&D), 1 μg/ml human LIF, 100 ng/ml Stem cell factor (SCF, 255-SC-010, R&D), 50 ng/ml EGF, and 10 μM Y27632 in Glasgow’s MEM (GMEM, 11710–035) [23]. After 4 days in the PGC induction medium, EB aggregates were fixed and stained for early PGC markers. Cell quantification of immunofluorescent staining was performed with Image J software normalized with the number of DAPI [24].

### Gene Expression Analysis

Total RNA was isolated by TRIzol^®^ reagent (15596–026) and treated with RNase-free DNase I (M6101, Promega, Madison, WI, USA) to remove genomic DNA. Treated RNAs were reverse-transcribed by random hexamer primers using the SuperScript III First-Strand Synthesis System (18080–051). qRT-PCR was conducted as previous described [22], with the CT value of *GAPDH* serving as the internal control. For endogenous and exogenous pluripotent gene analysis, primers were modified from Yu et al [20]. Primer sequences are listed in S1 Table.

### Statistics

For multiple group analysis, one-way ANOVA following Tukey's multiple comparisons test were used (GraphPad Software Inc., La Jolla, CA, USA).

## Results

### Derivation of hc-iPSCs Using Clinically Discarded CCs

**Fig 1:**
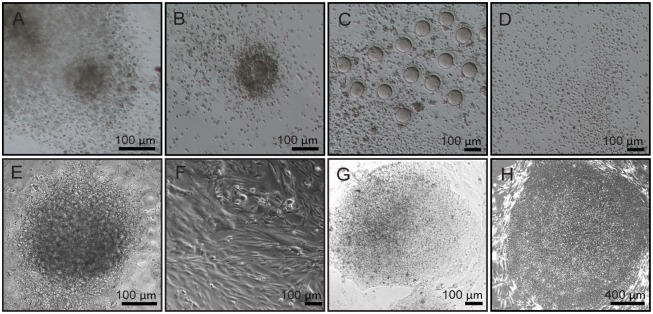
Derivation of hc-iPSCs. (A) Human cumulus-oocyte-complex (COC). (B) COCs treated with hyaluronidase for 1 minute. (C) Denudated oocyte. (D) Completely dissociated CCs. (E) Morphology of attached human CCs under phase contrast microscope. (F) Cultured CCs of spindle-like shape after passage. (G) Primarily formed iPSC colony after 25 days of induction. (H) hc-iPSCs after manually pick-up. Scale bar: 100 μm.

CCs were obtained from seven patients (Table 1) who had undergone ICSI at MMH IVF clinic with patients’ consent. Cumulus-oocyte-complex (COCs) were harvested (Fig 1A) and dissociated with hyaluronidase (Fig 1B). CCs denuded from multiple oocytes of the same patient were pooled (Fig 1C and 1D). Immediately after plating, CCs formed cluster structures (Fig 1E). These cells later formed flat monolayer (Fig 1F).

Approximately 25 days post lentiviral induction by *OCT4*, *SOX2*, *NANOG* and *LIN28*, cells with colony-like morphology appeared (Fig 1G). The induction duration is in line with iPSC derivation work using other types of donor cells, which is in the range of 3–4 weeks. Colonies were picked up and cultured in conventional hESM. Typical iPSC morphologies were observed (Fig 1H).

Out of seven patients, we successfully derived hc-iPSCs from Patient #1 and Patient #5, but not from other 5 patients. No correlation was detected between this success and available patient parameters, including age, reason for treatment, anti-Müllerian hormone (AMH) level, number of eggs retrieved, number of matured eggs, fertilization rate after ICSI, and pregnancy outcome. It is noted however, that both Patient#1 and #5 have their AMH levels in the low normal range (1.0–1.5 ng/mL), and no lines were derived from patients whose AMH levels are extremely low (0.5–1.0 ng/mL) or high (>4.0 ng/mL) [25–27]. To our knowledge, this is the first success in deriving patient-specific iPSCs using clinically discarded CCs.

**Fig 2:**
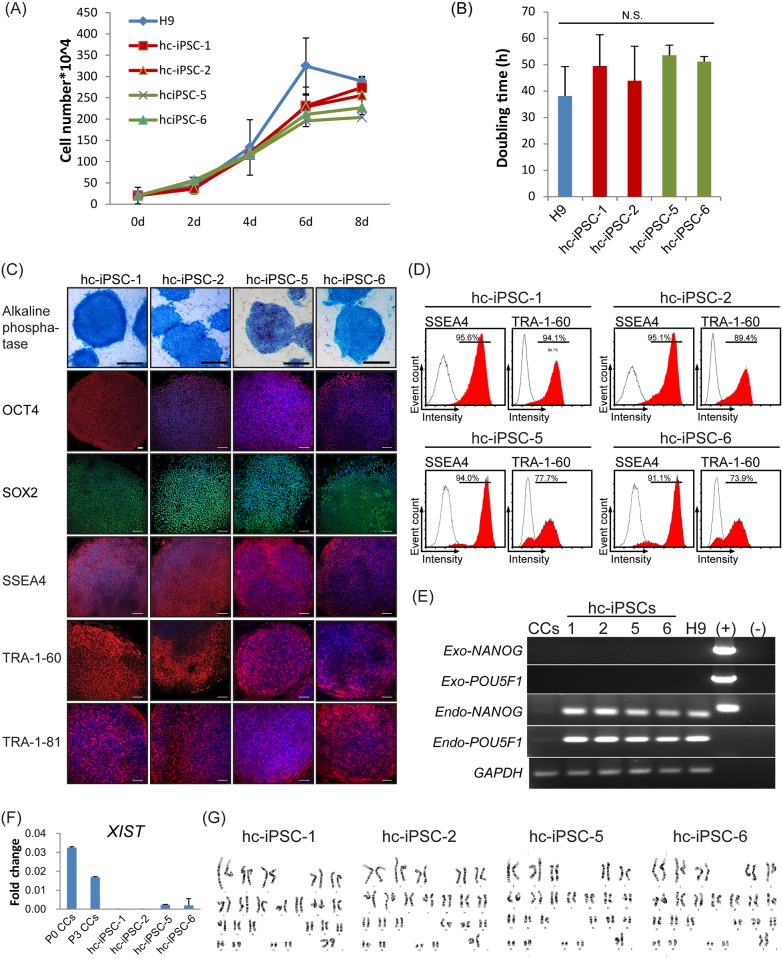
*In vitro* characterizations of hc-iPSCs. (A) The growth curve of hc-iPSCs (hc-iPSC-1, 2, 5, 6), and hESC H9 (3 replicates per line). (B) Population doubling time of hc-iPSC lines and hESC H9. (C) Alkaline phosphatase activity was detected in hc-iPSC-1, -2, -5 and -6 (Blue color, scale bar = 1 mm). Immunofluorescent with antibodies against OCT4, SOX2, SSEA4, TRA-1-60 and TRA-1-81 were also detected in hc-iPSC lines. Scale bar = 100μm. (D) Flow cytometry confirmed the percentage of SSEA4 and TRA-1-60 positive cells in hc-iPSC lines (red color), as the numbers on graphs indicated, respectively. Isotype controls were shown in white color. (E) Semiquantitative PCR for expression of pluripotent genes. Exogeneous *NANOG* and *POU5F1* (*Exo-NANOG*, *Exo-POU5F1*) genes were undetectable and endogeneous genes (*Endo-NANOG*, *Endo-POU5F1*) were expressed in hc-iPSC lines and hESC H9. cDNA of cumulus cells (CCs) at passage 3 was from mixture of patients. (+): template plasmids which containing *POU5F1* or *NANOG*. An unexpected band was amplified by endogeneous NANOG primer in *NANOG* plasmid with incorrect size of PCR product, which may due to sequence similarity. (-): negative control without template. *GAPDH* was used for internal control. (F) X chromosome inactivating transcripts *XIST* were detected in mixture of CCs at passage 0 (P0 CCs), and passage 3 (P3 CCs), but nearly undetectable in hc-iPSCs. (G) hc-iPSCs showed normal female karyotypes without chromosome translocation, deletion or replication.

Multiple iPSC lines were established for Patient #1 (hc-iPSC-1 to -4) and Patient #5 (hc-iPSC-5 to -8). Four lines (hc-iPSC-1, -2, -5 and -6) were randomly picked for long-term culture and characterization; all have reached Passage 24 or beyond to date. The growth curve and population doubling time of all hc-iPSC lines appeared like those of the human ES cell line H9 (Fig 2A and 2B). The other lines were frozen at Passage 5 to 10.

### The hc-iPSCs Express Typical Pluripotent Genes

Selected hc-iPSC lines at P10 to P15 were used for characterization assays. All lines expressed alkaline phosphatase (Fig 2C, top row). They expressed specific pluripotent markers, OCT4, SOX2, SSEA4 and TRA-1-60 and TRA-1-81, as determined by immunochemistry staining (Fig 2C, row 2–6). Consistently, high expression of SSEA4 and TRA-1-60 was confirmed by flow cytometry (Fig 2D).

One indicator of successful reprogramming of somatic cells to the pluripotency state is the activation of endogenous pluripotent genes [20]. Semi-quantitative PCR using primers targeting 3’ UTR of endogeneous *NANOG* and *POU5F1* revealed that endogenous *NANOG* and *POU5F1* were expressed in all hc-iPSC lines along with the silencing of the exogenous ones (Fig 2E).

### Both X Chromosomes are Activated in hc-iPSCs

Because hc-iPSCs were derived from female patients, we looked at the status of X chromosome (in)activation in the CCs and the derivative hc-iPSCs by measuring the expression levels of *XIST*, the long non-coding RNA (lncRNA) gene that is responsible for inactivating one X chromosome in differentiated cells, as previously described [28,29]. As expected, high *XIST* expression was present in CCs at P0 and P3; whereas extremely low *XIST* expression was found in the hc-iPSCs (Fig 2F), which indicates the active status of both X chromosomes. The karyotyping assay revealed that hc-iPSCs possess normal karyotype without chromosome translocation, deletion or replication (Fig 2G).

### The hc-iPSCs are Capable of Differentiating into All Three Germ Layers

**Fig 3:**
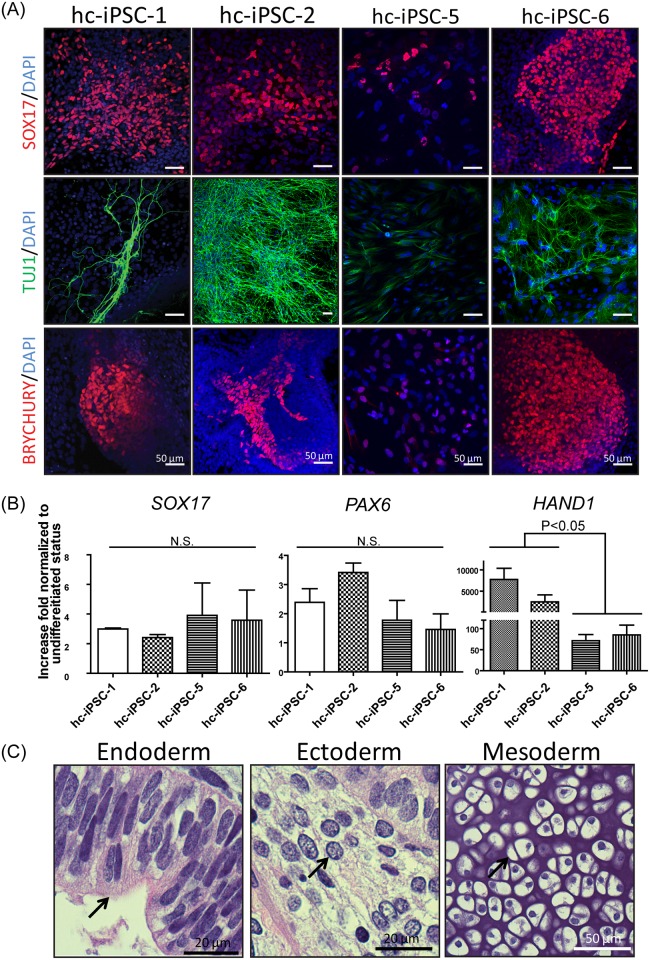
*In vitro* differentiation and teratoma assay. (A) The germlayer specific markers SOX17 (endoderm, red color), TUJ1 (ectoderm, green color), and the BRYCHURY (mesoderm, red color) were detected after *in vitro* differentiation in hc-iPSCs. DNA was stained by DAPI (blue). (B) Expression of germlayer specific genes (*SOX17* for endoderm, *PAX6* for ectoderm, and *HAND1* for mesoderm) were all upregulated in day 10 EBs compared with hc-iPSCs. (C) Ciliated epithelium (endoderm), neuronal-like (ectoderm) and cartilage (mesoderm) cells were present in hc-iPSC derived teratomas (stained with H&E). Arrows indicate corresponding cell types.

We next examined the differentiation capacity of hc-iPSCs. Following *in vitro* differentiation, we analyzed three germlayer markers, SOX17 (endoderm), BRYCHURY (mesoderm), and TUJ1 (ectoderm), on attached EBs by immunofluorescent staining (Fig 3A); *SOX17*, *HAND1*and *PAX6* were detected in 10-days-EBs by qRT-PCR (Fig 3B). As expected, all hc-iPSC line cells are capable of differentiating into all three germlayers.

In line with the *in vitro* results, teratoma analysis demonstrated that hc-iPSCs possess the capacity for forming endoderm (ciliated epithelium), mesoderm (cartilage), and ectoderm (neuron) after transplantation to immunodeficient mice (Fig 3C).

### Hc-iPSCs are capable of differentiating to PGCLCs

Considering the close relationship between CCs and the oocytes, we hypothesize that hc-iPSCs may be a good source for germ cell derivation, such as primordial germ cells (PGCs). As the first step to demonstrate this feasibility, we worked to differentiate hc-iPSCs to PGCLCs, following a published protocol [23]. For comparison, female (iPSC0102) and male (iPSC0207) iPSC lines derived from PBMCs and female hESC line H9 were also used for PGCLC differentiation.

**Fig 4:**
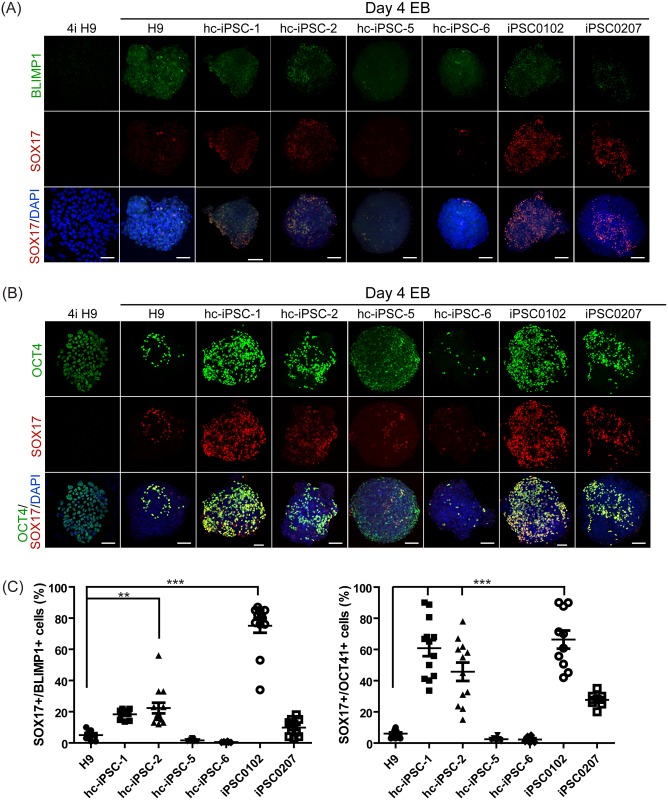
PGCLCs derived from hc-iPSCs. Immunofluorescent staining of (A) BLIMP1 (green color) and (B) OCT4 (green color) in 4-day-old EBs cultured with PGC induction medium on hc-iPSC-1, -2, -5 and -6 (third to fifth column), PBMC derived iPSC0102 and iPSC0207 (right two column). H9 hESCs (left column) adapted in 4i medium was also detected for BLIMP1 and OCT4 antibodies. Noted that there was no BLIMP1 signals detected in undifferentiated H9 in 4i condition. All samples were doubly stained with SOX17 antibody (red color) sequentially. DAPI was used for counterstain (blue). Scale bar = 50μm. (C) Quantification of immunofluoresent staining of PGCLCs. BLIMP1/SOX17 and OCT4/SOX17 double positive cells were counted for PGCLCs and normalized with the number of DAPI. Percentage of PGCLCs per EB of all lines was presented and significance was compared with control hESC H9. ** indicates P<0.005. *** indicates P<0.001.

BLIMP1 (also known as PR domain-containing 1, PRDM1) is critical for primordial germ cell formation in mouse and in humans [23]. SOX17 coordinates with BLIMP1 for PGC specification and is reported to be indispensable and sufficient for PGCLC induction from human competent pluripotent stem cells [23]. SOX17/BLIMP1 or SOX17/OCT4 can be used as makers for PGCLCs [23]. Here we looked at SOX17 profile along with that of OCT4 or BLIMP1 in day 4 EBs after PGC induction by immunofluorescent staining. Putative PGCLCs, judged by double positive SOX17/OCT4 or SOX17/BLIMP1, were present in EBs derived from hc-iPSCs (-1, -2, -5, -6), hb-iPSCs (iPSC0102, iPSC0207) and hESCs (H9). It appears that highest amount of BLIMP1 containing putative PGCLCs are present in iPSC0102, followed by hc-iPSCs-1, -2 (from Patient #1) and iPSC0207, and lowest in H9, hc-iPSC-5 and -6 (from Patient #5) (Fig 4A). Similar expression levels of OCT4/SOX17 among different lines were observed (Fig 4B). The quantitative result of immunofluorescent staining was determined in Fig 4C. H9 maintained in 4i culture (i.e. without subsequent PGCLC induction treatment) was used as the negative control of BLIMP1 and SOX17, and the positive control for OCT4 (Fig 4A and 4B, left panel).

These data show that hc-iPSCs are capable of differentiating to PGCLCs, a critical link towards the ultimate goal of *in vitro* generation of gamete cells.

## Discussion

CCs are by-products of human infertility treatments. In a routine ICSI procedure, these cells are stripped from metaphase II (MII) oocytes and discarded. Limited efforts have been taken to make use of clinically discarded CCs. In one direction, gene expression patterns in CCs were used to predict the oocyte quality and consequently the developmental competency of the derivative embryos [30].

In animals, CCs were considered among the “easiest” for NT based reprogramming, evidenced by large number of cloned animals derived from CCs in a variety of species including mouse and cattle with relative high efficiencies as compared to those using other types of donor cells such as fibroblast cells [15,31,32].

Surprisingly however, there are few reports on utilizing CCs for iPSC derivation. Multiple factors may have contributed to the few success of using human CCs for iPSC derivation: (i) in NT based reprogramming work, only one donor cell is required for generating a cloned embryo. In iPSC derivation work, however, large number of good quality cells is needed, which is often not the case when CCs from one individual undergoing infertility treatment are used. It has been reported that on average there are only ~16,000 cells surrounding one egg retrieved in IVF clinics [33], i.e. even in the patient who had the most number (Table 1, Patient#4, n = 20) of eggs retrieved in the present work, there were only ~320,000 CCs; (ii) derivation of female iPSCs is known to require extra efforts comparing to their male counterparts, because there is the extra X chromosome in female cells to be reprogrammed [34]; and (iii) most CCs are from patients undergoing infertility treatment. It is not known whether or not the hormone treatment to the patient and/or the infertility itself make these CCs suboptimal for iPSC reprogramming.

The present work proves that clinical discarded CCs can be used for patient-specific iPSC derivation, albeit of low efficiency. Once established, these hc-iPSCs are similar to iPSCs derived from other types of donor cells in all aspects that were characterized, importantly, including possessing the capacity of differentiating to all three germ layers both *in vitro* and *in vivo*.

The success/failure of hc-iPSC derivation does not correlate with the age of the patient, reason for infertility treatment (e.g. female factor or male factor), the number of eggs retrieved, the fertilization rate, or the pregnancy outcome among these seven patients. Increasing patient number may help identify contributing factors to such variation. On the other hand, this indicates that the current protocol is not universally effective on reprogramming CCs of different patients. In the present study, we used the lentiviral mediated OSNL factors for reprogramming, as this would enable generating a good quality of human iPSC in previously developed system [20]. Future work should be dedicated to optimize the protocols to allow efficient derivation of hc-iPSCs from most, if not all, patients.

In the context of human infertility applications, we attempted to derive PGCLCs using hc-iPSCs. Derivation of PGCLCs represents the first step of artificial gametes production, i.e. formation of the male or female PGCs. In 2012, Hayashi et al from Kyoto University, Japan reported the first successful case of deriving oocytes-like cells (OLCs) from iPSCs (and ESCs) in a mouse model, in which PGCLC derivation was achieved *in vitro*, followed by transplantation of the PGCLCs to female gonads to form OLCs *in vivo* [35]. In 2014, Irie et al used a similar strategy to achieve high efficiency PGCLC derivation using human pluripotent stem cells [23]. Given the close physical and developmental associations of CCs and oocytes, we hypothesized that the hc-iPSCs may encounter fewer epigenetic barriers on route towards PGCs as compared to iPSCs derived from other cell types such as those from blood cells (i.e. hb-iPSCs). Indeed two (hc-iPSC-1 and -2) out of four hc-iPSC lines tested resulted in the high percentage of SOX17/BLIMP1 or SOX17/OCT4 double positive cells, higher than those from hESC line H9. However, the other two lines hc-iPSC-5 and -6 led to the low percentage of putative PGCLCs. It is noted that hc-iPSC-1 and -2 are from Patient#1 while hc-iPSC-5 and -6 are from Patient#5, indicating quality variations between hc-iPSC lines of different patient source. It remains to be tested whether hc-iPSCs possess better chances towards the ultimate gamete, more likely oocyte formation.

## Conclusions

The present work demonstrates that clinically discarded CCs can be used to establish patient-specific iPSCs. These cells possess the capacity to differentiate into all three germlayers and represent a potential source for gamete derivation. Our work expands the available biomaterials for regenerative medicine.

## Supporting Information

S1 Table Primer Sequence. The primers used for qRT-PCR and semi-quantitative PCR were listed. Related to Figs 2 and 3.
(DOCX)
