# Supplementary material for: Derivation of Patient Specific Pluripotent Stem Cells Using Clinically Discarded Cumulus Cells
Source: PLoS One. 2016 Nov 1;11(11):e0165715. doi: 10.1371/journal.pone.0165715 (PMC5089679; doi:10.1371/journal.pone.0165715)
Supplement: S1 Table — The primers used for qRT-PCR and semi-quantitative PCR were listed. Related to Figs 2 and 3. (DOCX) [file pone.0165715.s001.docx]

S1 Table. Primer sequences.

| Gene | Primer Sequence |
| --- | --- |
| Endo-POU5F1 | F: AGTTTGTGCCAGGGTTTTTG  R: ACTTCACCTTCCCTCCAACC |
| Endo-NANOG | F: TTTGGAAGCTGCTGGGGAAG  R: GATGGGAGGAGGGGAGAGGA |
| SOX2 | F: TGCTGCCTCTTTAAGACTAGGAC  R: CCTGGGGCTCAAACTTCTCT |
| SOX17 | F: ACGCCGAGTTGAGCAAGA  R: GTGCAGGAAGCCGCCCTC |
| PAX6 | F: TCACCATGGCAAATAACCTG  R: CAGCATGCAGGAGTATGAGG |
| XIST | F: AGGGAGCAGTTTGCCCTACT  R: CACATGCAGCGTGGTATCTT |
| Exo-NANOG | F:ATGCCTCACACGGAGACTGT  R: AGAGGAACTGCTTCCTTCACGACA |
| Exo-POU5F1 | F: TCAAGCCTCAGACAGTGGTTC  R: CGGTTACAGAACCACACTCG |
| GAPDH | F: AGGGCTGCTTTTAACTCTGGT  R: CCCCACTTGATTTTGGAGGGA |
